# Frames of reference in small-scale spatial tasks in wild bumblebees

**DOI:** 10.1038/s41598-022-26282-z

**Published:** 2022-12-15

**Authors:** Gema Martin-Ordas

**Affiliations:** 1 Department of Psychology, University of Oviedo, Oviedo, Spain; 2 Division of Psychology, University of Stirling, Stirling, UK

**Keywords:** Animal behaviour, Experimental evolution

## Abstract

Spatial cognitive abilities are fundamental to foraging animal species. In particular, being able to encode the location of an object *in relation to* another object (i.e., *spatial relationships*) is critical for successful foraging*.* Whether egocentric (i.e., viewer-dependent) or allocentric (i.e., dependent on external environment or cues) representations underlie these behaviours is still a highly debated question in vertebrates and invertebrates. Previous research shows that bees encode spatial information largely using egocentric information. However, no research has investigated this question in the context of relational similarity. To test this, a spatial matching task previously used with humans and great apes was adapted for use with wild-caught bumblebees. In a series of experiments, bees first experienced a rewarded object and then had to *spontaneously* (Experiment 1) find or *learn* (Experiments 2 and 3) to find a second one, based on the location of first one. The results showed that bumblebees predominantly exhibited an allocentric strategy in the three experiments. These findings suggest that egocentric representations alone might not be evolutionary ancestral and clearly indicate similarities between vertebrates and invertebrates when encoding spatial information.

## Introduction

Spatial cognitive abilities are fundamental to foraging animal species, and research has shown that different species share similar spatial abilities. For example, the elementary abilities that contribute to navigation (e.g., path integration) have been found both in vertebrates (e.g., birds rodents^1,2^) and invertebrates (e.g., ants, bees^3–5^).

Being able to encode the location of an object *in relation to* another object is critical to establish *spatial relationships*^6,7^. The representation of these spatial relationships follows frames of reference (FoR)—i.e., means of representing the directional relationship between objects in a specific space and in relation to a shared referential object (e.g., the car is *in front of* the house^8^). When representing spatial relationships, humans use different FoRs: (1) *egocentric* FoRs refer to the location of an object relative to the speaker (e.g., the car is on my right); (2) *object-centred* FoRs refer to locations relative to a landmark (e.g., the car is in front of the house); and (3) *geocentric* FoRs refer to locations relative to a global frame (e.g., the car is South-West of the house).

Which FoRs humans use vary with language and culture^8,9^. In a study conducted by Haun et al.^9^, participants were presented with an array of five identical cups on a table and observed an experimenter hiding an object in one of the cups. Participants were then moved around and experienced a second identical array of cups on the table, but from the opposite perspective. They were asked to find a second hidden object, based on the location of the first hidden one. The results showed that while Dutch speakers relied on egocentric FoRs—which is consistent with their egocentric linguistic pattern-, Haijjom speakers relied on geocentric ones—which is consistent with their geocentric linguistic pattern. More interestingly, when presenting the same task to young children and great apes, both performed better when the task required the use of allocentric FoR (which includes geocentric and object-centred FoRs) than egocentric ones. These findings indicate that there is a shared bias towards allocentric encoding of spatial relationships with humans and great apes’ common ancestor. However, this bias can be overridden by culture and language^9,10^. Other studies with vertebrates have also shown a preference for allocentric over egocentric strategies^11–13^.

Research with invertebrates has shown that even though they can use both strategies^14–17^, they have preferences for egocentric over allocentric strategies^18–20^. However, none of these experiments with invertebrates used a relational paradigm. Importantly, being able to successfully forage in a complex environment involves establishing relationships between different objects (e.g., the purple flower is *next to* the yellow flower).

Most of the evidence on relational processing (i.e., acquiring and extrapolating implicit knowledge) and relational learning (e.g., learning same/different, larger/smaller rules) comes from honeybees^21–23^, though bumblebees have recently been shown to spontaneously attend to relational similarity^24^. Additionally, bumblebees have extraordinary spatial memory skills^25,26^. Therefore, they are good candidates to investigate their spatial cognitive strategies in a relational task. To this purpose, I modified the FoR paradigm (i.e., spatial relational task) developed by Haun et al.^9^ for use with wild-caught bees. Specifically, in a task involving small-scale spatial relations I investigated whether bumblebees show preferences for encoding spatial relations in particular FoRs (Experiment 1) and whether they can acquire alternative ones (Experiments 2 and 3).

Spatial cognition and relational reasoning are critical to human cognition. Whereas the former facilitates encoding spatio-temporal information^7^, relational reasoning plays a pivotal role in establishing abstract thinking and generalizing information across different contexts^6,27^. Comparative evidence indicates the existence of an inherited bias toward allocentric FoRs in human and non-human primates. Investigating whether the preference for environment (i.e., allocentric FoR) rather than self-centred (i.e., egocentric FoR) spatial relations extends to invertebrates is critical to understand the structure of spatial relational thought. If a shared structure exists, it is expected that, like in vertebrates, bees would show a bias for allocentric encoding of spatial relationships.

### Experiment 1: spontaneous searching strategies

**Figure 1:**
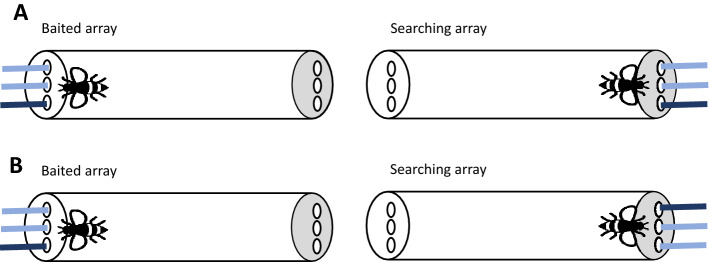
Bird’s eye view of the experimental setup for Experiments 1–3. Bees experience one of the three paper strips baited on the transparent side (Baited array), and they had to search among the strips on the opaque side (Searching array). All the stimuli were identical. Note that the darker blue colour has only been used to represent an Allocentric searching strategy (**A**) and an Egocentric searching strategy (**B**).

Here I examined whether bumblebees *spontaneously* show a preference for egocentric or allocentric searching strategies. To do so, I followed a similar procedure to Martin-Ordas^24^. Bumblebees experienced two sets of objects (Baited and Searching arrays). First, they were presented with three Baited objects—only one of them was dipped in sucrose. Their task was to find the corresponding object in the Searching array (see Fig. 1). The two sets of three identical objects were placed in a straight line and allowed either the use of egocentric or allocentric strategies. If bees show a bias for egocentric strategies, in the Searching array they should select the object that maintains the same position relative to their body axis as in the Baited array. If their bias is towards allocentric strategies, then in the Searching array bees should select the object that maintains the same position relative to a salient landmark (e.g., experimenter, other strips) as in the Baited array.

#### Subjects

The data was collected between March and June 2022 in Northumberland and Stirlingshire (UK). The two areas where the bees were caught were separated by more than 100 km. A total of 25 bees was captured, although 3 completed less than 6 trials and were not included in the analyses. The final sample was integrated by 22 bees of the following species: *Bombus pratorum* (n = 8), *Bombus pascuorum* (n = 7), *Bombus terrestris* (n = 2), *Bombus lapidarious* (n = 1), *Bombus lucorum* (n = 2), *Bombus bohemicus* (n = 1) and *Bombus hypnorum* (n = 1). Sex was visually identified (females = 21 and 1 could not be clearly identified).

#### Apparatus

A transparent plastic tube (14 × 3.5 cm) with 3 holes at one end (transparent end) and 3 at the other end (lid end) through which the stimuli could be inserted was used (see Fig. 1A,B). The distance between the holes—for transparent and lid ends- was equal (1 cm). Blue strips of paper (3 × 0.2 cm) were used as stimuli: three were introduced through the transparent end of the tube (Baited array) and three through lid end of the tube (Searching array). The strips were fixed in playdoh to introduce them simultaneously in the tube.

#### Procedure

Once the bees were captured, they were left in the tube on average for 2 h prior to testing. This was done to allow the bees to habituate to the tubes and become motivated to forage^28^. Bees were tested in the field on a T-shaped platform. Experiments were always conducted in the morning. On one of the sides of the platform 5 holes were drilled (see Fig. S1 Supplementary Materials). The experimenter (E) sat in front of the platform and the tubes were inserted through the holes so that the transparent end always faced the E. Playdoh containers were placed on both sides of the tube so the same environmental elements were present for all bees. Subjects were first presented with the Baited strips on the transparent side of the tube. Only one of the strips—left, middle or right (E’s perspective)- was dipped in 50% (w/w) sucrose. Bees were allowed to explore the 3 strips. Once the bee made contact with the strip dipped in sucrose—either by using its antennae or proboscis- it was given (on average) 5-6 s to drink the solution. Then, the Baited strips were removed, and the E introduced the Searching strips through the lid end. These strips were dipped in water. Note that it is common to use distinctive Baiting and Searching arrays in spatial matching tasks^29^. A choice was considered when bees touched the strip with the antennae or proboscis. Each bee received a total of 12 trials and the position of the reward in the Baited strips was counterbalanced across trials. New paper strips were used for each trial and in each array. The inter-trial-intervals were approximately 1 min for each bee. During this time, subjects were allowed to freely move in the tube. Importantly, bees did not receive any training prior to these trials and their choices were not rewarded. After the experiment was over and before releasing them, bees were individually marked. Posca markers were used for this purpose.

#### Analyses

Data were analysed with R version 2022.07.0 using non-parametric t-test (Wilcoxon). Percentage of “allocentric” and “egocentric” responses were compared. Middle trials were analyzed separately because the use of both strategies—allocentric and egocentric- led to the same solution. Wilcoxon tests were used to analyse if performance was significantly above chance. *P* values below 0.050 were considered to provide evidence for significant differences.

### Results and discussion

**Figure 2:**
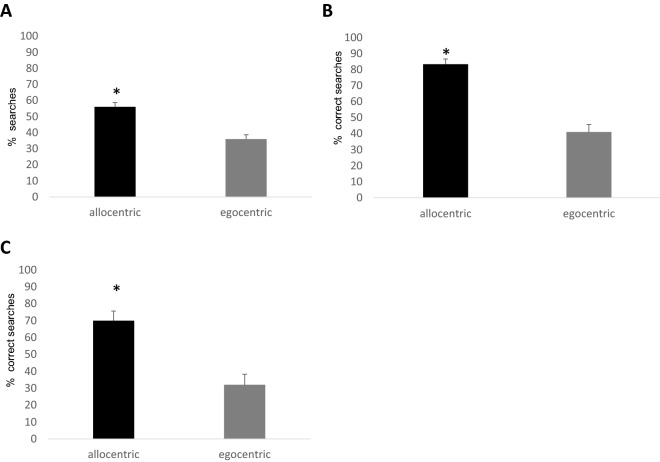
(**A**) Mean percentage of spontaneous allocentric and egocentric searches in Experiment 1. (**B**, **C**) The mean percentage of correct responses in Experiments 2 and 3. The asterisk indicates the conditions in which bees performed significantly above chance. Bars represent standard errors.

There were differences between subjects’ searching strategies (W = 13, *P* = 0.002, see Fig. 2A)—with bumblebees more frequently selecting the strips following an allocentric strategy than the strips following an egocentric strategy. In fact, subjects used the allocentric searching strategy significantly above chance (W = 253, *P* < 0.001), but not the egocentric one (W = 141, *P* = 0.380). Similarly, subjects chose the correct strip in the *Middle* trials significantly above chance (*P* < 0.0001; mean = 68.56).

The results of this experiment indicate that when processing small-scale spatial relations between objects, bumblebees *spontaneously* rely more on environmental cues than self-centred ones. Crucially, previous research has shown that, when confronted with a spatial relational *learning* paradigm—in which *both* egocentric and allocentric strategies were trained- young children and great apes still showed a preference for allocentric compared to egocentric strategies^9^. In Experiment 2, a variation of the spatial relational paradigm was used to test whether bees could acquire both searching strategies.

### Experiment 2: learning allocentric and egocentric strategies

As before, bumblebees were presented with two sets of 3 objects each (Baited and Searching arrays). Following Haun et al.^9^, there were two conditions—*Allocentric* and *Egocentric-* in which only correct responses were rewarded. In the *Egocentric* condition, the correct strip in the Searching array kept the same position in relation to the subject’s body axis as in the Baited array (see Fig. 1A,B). In the *Allocentric* condition, the correct strip in the Searching array kept the same position in relation to a salient landmark or the surrounding environment as in the Baited array. It was predicted that if bees can acquire both strategies, no difference in performance between the *Allocentric* and *Egocentric* conditions would be found. However, if bees—like children and great apes- have a preference for one strategy over the other, regardless of the learning experience, then they would struggle with the *Egocentric* condition.

#### Subjects

The data was collected between March and June 2022 in Northumberland and Stirlingshire (UK). A total of 25 bees was captured, however 4 failed to complete all the trials for both conditions and were not included in the analyses. The final sample consisted of 21 bees of the following species: *Bombus pascuorum* (n = 13), *Bombus hypnorum* (n = 2), *Bombus pratorum* (n = 2), *Bombus bohemicus* (n = 1), *Bombus hortorum* (n = 1), *Bombus lucorum* (n = 1) and *Bombus terrestris* (n = 1). Sex was visually identified (females = 19 and 2 could not be clearly identified).

#### Apparatus

The same tubes and strip sizes as in Experiment 1 were used.

#### Procedure

As before, bees were left in the tube on average for 2 h prior to testing. The *Allocentric* and *Egocentric* conditions were administered in a block of 12 randomized trials for each subject, counterbalanced for order across subjects. The transition between the conditions was unmarked. Two trials, in which the middle strip was rewarded in the Baited array (*Middle* condition), were randomly intermixed within the blocks. In these middle trials, both allocentric and egocentric rules led to the same solution. Thus, each bee received a total of 14 trials—6 *Allocentric*, 6 *Egocentric* and 2 *Middle* trials- and for the *Allocentri*c and *Egocentric* trials the position of the reward—left or right- was counterbalanced across trials. In the Searching array, only one of the strips was dipped in sucrose and the other two were dipped in water. The same procedure as in Experiment 1 was followed for administering the different conditions. A choice was considered when the bees touched the strip with the antennae or proboscis and they were only allowed to make one choice. New paper strips were used for each trial and in each array. Importantly, bees did not receive any training prior to these trials.

#### Analyses

Subjects were excluded following Haun et al.’s^9^ criteria. Bees that did not perform at least 50% correct in the *Middle* condition were excluded from the analyses (n = 3). In the *Allocentric* and *Egocentric* conditions, bees had 33% chances of choosing a Searching strip that was not related to a Baited strip by not following an egocentric or allocentric strategy. Since none of the bees did so 50% of the times or more, no bees were excluded from the final analysis. Thus, the final sample was n = 18. The comparison between the *Egocentric* and *Allocentric* conditions was analysed with R version 2022.07.0 using a binomial general linear mixed model (GLMM)^30^. The dependent variable was whether bee’s choice was correct (coded 1) or incorrect (coded 0), the independent variable was experimental condition as a categorical variable and a random factor was the individual bees. A second model was run including bee’s choice as dependent variable, experimental condition, order of administration of experimental conditions and location of rewards as independent variables and individual bees as a random factor (see Supplementary Materials). Wilcoxon tests were used to analyse if performance in each condition was significantly above chance. *P* values below 0.050 were considered to provide evidence for significant differences.

### Results and discussion

There was an effect of condition in bees’ performance (estimate *SD* = -1.816,* z* = -5.62, *P* < 0.001, 95% CI = 0.086 to 0.307; Fig. 2B)—bees performed better in the *Allocentric* condition than in the *Egocentric* condition. As in Experiment 1, subjects performed significantly above chance in the *Allocentric* (Wilcoxon test: *W* = 231, *P* < 0.001) but not in the *Egocentric* condition (Wilcoxon test: *W* = 54, *P* = 0.065; see Supplementary Materials for individual performances Table S2 and learning curves Fig. S2).

In the present spatial relational paradigm, bumblebees also showed a bias towards the use of allocentric strategies. These results are along the same lines to those reported by Haun et al.^9^ with children and great apes. It is important to note, though, that bees received fewer trials compared to subjects in Haun et al.’s study. Thus, one could speculate that had bees been provided with more trials, their performance in the *Egocentric* condition could have been more robust. Note that while 43% of the bees showed a preference for egocentric strategies in the *Egocentric* condition, all of them consistently used allocentric strategies in the *Allocentric* condition. Remarkably, the number of trials used in the present paradigm sufficed to improve bees’ performance in the *Allocentric* condition—recall that in Experiment 1 bees’ mean response was 56% for this condition and 83% in Experiment 2 (Fig. 2B). It is possible that visual or/and odour cues (associated with the sucrose but not water) in the Searching array might have contributed to increase subjects’ bias towards allocentric strategies in this condition^31,32^. If this were the case, then performance should have also improved in the *Egocentric* condition. However, this is not what was found.

There is a second difference between Haun et al.^9^’s paradigm and the present one: whereas in the incorrect trials Haun et al. showed subjects under which cup the reward was hidden, in the current experiment bees were not given that information. That is, if bees made an incorrect choice, they were not allowed to find the rewarded strip. One could argue that seeing where the correct reward was hidden could have facilitated the learning experience. Haun et al.’s results showed, though, that apes did not learn to use the egocentric strategy in those circumstances. However, since bees were only allowed to explore one single strip, it is still possible that their spontaneous preference towards allocentric strategies was reinforced—this being the reason why a higher percentage of searches in the *Allocentric* condition was found. To control for this possibility, a modified spatial relational learning paradigm was conducted.

### Experiment 3: learning allocentric and egocentric strategies (II)

Bees were presented with a modification of the spatial relational paradigm used in Experiment 2. The main structure of the experiment was preserved; that is, bees experienced both the *Allocentric* and *Egocentric* conditions. However, now bees received a series of training trials—in which the Searching array contained one strip dipped in sucrose- followed by a series of experimental trials—in this case, the strips in the Searching array were dipped in water. The predictions were the same as for Experiment 2.

#### Subjects

The data was collected between June and August 2022 in Stirlingshire (UK). A total of 27 bees was captured, however 2 failed to complete all the trials for the *Allocentric* and *Egocentric* conditions and 5 did not complete the *Middle* trials. Thus, the final sample included 20 bees: *Bombus lucorum* (n = 10), *Bombus terrestris* (n = 5), *Bombus pascuorum* (n = 4) and *Bombus hortorum* (n = 1). Sex was visually identified (females = 19, males = 1).

#### Apparatus

The same tubes and strip sizes as in Experiments 1 and 2 were used.

#### Procedure

After an average of 2 h in the tubes, bees were presented with the *Allocentric* and *Egocentric* conditions. The two conditions were administered in 18 trials for each subject—6 training trials per condition followed by 2 experimental trials per condition- counterbalanced for order across subjects. In the training trials, only one of the Searching strips—left or right (E’s perspective)- was dipped in sucrose and which strip was baited depended on the condition (see Fig. 1). Importantly, in the experimental trials the 3 Searching strips were dipped in water. The transition between the conditions and type of trials was unmarked. After the 16 trials, bees were presented with 2 trials in which the middle strip was rewarded in the Baited array (*Middle* condition) and the 3 Searching strips were dipped in water.

A procedure similar to Experiments 1 and 2 was used to administer the conditions. A choice was considered when the bees touched the strip with the antennae or proboscis. In the training trials, subjects were allowed to make as many searches as needed until they found the rewarded strip. In the experimental trials, only one search was allowed. New paper strips were used for each trial and in each array. Bees did not receive any training prior to these trials.

#### Analyses

None of the subjects were excluded following Haun et al.’s criteria (e.g., low performance in the *Middle* trials). Data were analysed with R version 2022.07.0 using a binomial general linear mixed model (GLMM)^30^. Only experimental trials were included in the analyses. The dependent variable was whether bee’s choice was correct (coded 1) or incorrect (coded 0), the independent variable was experimental condition—*Allocentric* and *Egocentric*- as a categorical variable and a random factor was the individual bees. A second model was run including the bee’s choice as dependent variable, experimental condition, order of administration of experimental conditions and location of rewards as independent variables and individual bees as a random factor (see Supplementary Materials). Wilcoxon tests were used to analyse if performance in each condition was significantly above chance. *P* values below 0.050 were considered to provide evidence for significant differences.

### Results and discussion

As before, bees’ responses were determined by condition (estimate *SD* = -1.572,* z* = -3.269, *P* = 0.001, 95% CI = 0.080 to 0.573; Fig. 2C) and bees performed better in the *Allocentric* compared to the *Egocentric* condition. Similar to Experiments 1 and 2, subjects performed significantly above chance in the *Allocentric* (Wilcoxon test: *W* = 210, *P* < 0.001) but not in the *Egocentric* condition (Wilcoxon test: *W* = 84, *P* = 0.434; see Supplementary Materials for individual performances Table S3 and learning curves Fig. S3). When performance in the *Middle* trials was analysed, subjects chose the correct strip significantly above chance (Wilcoxon test: *W* = 210, *P* < 0.001; mean = 75).

The results replicated those from Experiment 2; that is, in a modified spatial relational learning paradigm, bees showed a bias towards allocentric searching strategies. Bees still performed significantly above chance in the *Allocentric* condition, however, compared to Experiment 2, their performance dropped in both *Allocentric* and *Egocentric* conditions. Thus, it is possible that performance in Experiment 2 was driven by the reinforcement of their preferred strategy. Still individual performance indicated that while 40% of the bees followed an allocentric strategy in both experimental trials of the *Allocentric* condition, only 10% followed an egocentric strategy in the experimental trials of the *Egocentric* condition. Irrespective of what factors might have influenced bumblebees learning experience, what it is clear from these findings is that, when processing small-scale relations between objects, bees rely on environmental cues rather than self-centred ones.

## General discussion

Taking Experiments 1–3 together, the results show that wild-caught bumblebees prefer to process small-scale spatial relationships based on environmental cues as shared reference between objects. These findings indicate that invertebrates’ spatial relational cognition is not necessarily egocentric and, importantly, that bumblebees share the structure of spatial relational thought with primates.

Allocentric strategies offer the advantage of being able to locate new points (e.g., food sources) from different locations if the external cues (e.g., stationary objects) remain the same. Thus, in habitats where landmarks are easy to distinguish and reliable, an allocentric strategy is more effective^20^. However, in contexts where landmarks are hard to distinguish or ephemeral and, therefore, unreliable, then the use egocentric strategies is more advantageous^33^. The fact that bees did not learn to consistently use egocentric strategies in the present paradigm should be carefully considered. First of all, this does not mean that bees do not deploy egocentric strategies—in fact, a number of bees in Experiments 2 and 3 did use egocentric strategies. Second, it is arguable that the number of trials in Experiments 2 and 3 was not sufficient for bees to learn to use egocentric strategies. However, previous studies^28^ have shown that bees can learn associative rules using similar number of trials. Still, the cognitive skills assessed in those studies and the present ones are different; thus, it is possible that if bees had been given more trials, they might have been able to consistently use egocentric strategies. It may also be argued that training bees to use a strategy that they already favour (i.e., allocentric) does not leave much room for improvement and, thus, training should have focused on the use of the egocentric strategy. Although bees performed better in the *Allocentric* compared to the *Egocentric* condition, their performance never reached ceiling levels in the *Allocentric* conditions. However, it is possible that training both strategies in the same sessions might helped to establish one of the strategies—which might have interfered with the learning of the other one.

Previous research has shown that in order to find food resources or their nest bees and ants encode the appearance of the landmark and, later, “match” the stored “snapshot” (i.e., “snapshot matching”) with the current visual input^3,34,35^. If bees in the present studies had used the snapshot matching strategy, egocentric strategies would have been the predominant strategy. However, the results show the opposite pattern—allocentric strategies were consistently used across the 3 experiments. It is also likely that the environment in which bees were tested favoured the use of these allocentric strategies. Research has shown that an environment rich in visual cues and landmarks biases animals towards using allocentric strategies^36^. However, since in the current experiments both conditions were conducted in similar environments, this explanation could not account for bees’ preference for the allocentric strategies.

The implications of the present findings add to the debate on the types of representations underlying spatial cognition. As previously mentioned, Haun et al.^9,10^ suggest that allocentric encoding is phylogenetically ancestral and, thus, shared among different animal species. It is the human-specific cultural influences that override the allocentric biases so that some human cultures show a bias towards egocentric strategies. An alternative view argues that human and non-human animals primarily rely on egocentric representations and that uniquely human forms of spatial representation (e.g., geometric information) build on these egocentric representations as well as on spatial language^37^. A third approach proposes that egocentric and allocentric strategies can co-exist in parallel^38–40^. The results presented here support the former view—bumblebees showed a clear bias towards allocentric strategies. However, a small number of bees were able to use both strategies—suggesting that allocentric and egocentric strategies might co-occur in these insects. Based on these findings, one could suggest not only that the egocentric strategies alone may not be evolutionarily ancestral, but also that culture and language might not be required to flexibly use different spatial frames of reference^41^.

In summary, bumblebees, like non-human primates, show a bias towards the use of allocentric frames of reference in the context of small-scale spatial relations. When trained to use both strategies, bees still showed a preference for the allocentric strategies, although a few individuals were able to also use egocentric strategies. While these results suggest that allocentric strategies are evolutionarily ancestral, tantalizingly they also indicate that both strategies could co-exist in bees. Studies like the ones presented here are critical to understand the evolution of spatial cognition. Theories of human spatial cognition will benefit from integrating these findings in order to draw inferences not only about what traits are unique to humans but also about the role that language might play in the evolution of spatial cognition.

## Supplementary Information

Supplementary Information 1.

Supplementary Information 2.

## Data Availability

All data generated or analysed during this study are included in this published article (and its supplementary information files).
