# Supplementary material for: Frames of reference in small-scale spatial tasks in wild bumblebees
Source: Sci Rep. 2022 Dec 15;12:21683. doi: 10.1038/s41598-022-26282-z (PMC9755249; doi:10.1038/s41598-022-26282-z)
Supplement: Supplementary file 1 — Supplementary Information 1. [file 41598_2022_26282_MOESM1_ESM.docx]

**Supplementary Materials**

Searching array

Baiting array

**Figure S1**. T-shaped platform and experimental setup used for the present experiments

**Experiment 1**

**Table S1**. Individual performance in Experiment 1: Spontaneous searching strategies

| Subject | % allocentric searches | % egocentric searches | Species |
| --- | --- | --- | --- |
| 1 | 37.5 | 62.5 | *B. pratorum* |
| 3 | 50 | 37.5 | *B. pratorum* |
| 5 | 50 | 33.33 | *B. lapidarious* |
| 6 | 50 | 33.33 | *B. pratorum* |
| 13 | 62.5 | 37.5 | *B. pascuorum* |
| 14 | 37.5 | 37.5 | *B. pratorum* |
| 16 | 87.5 | 12.5 | *B. pratorum* |
| 19 | 62.5 | 37.5 | *B. pascuorum* |
| 21 | 62.5 | 37.5 | *B. pratorum* |
| 23 | 50 | 37.5 | *B. pascuorum* |
| 27 | 75 | 25 | *B. pascuorum* |
| 28 | 50 | 50 | *B. pascuorum* |
| 30 | 62.5 | 37.5 | *B. pratorum* |
| 31 | 62.5 | 25 | *B. bohemicus* |
| 34 | 50 | 37.5 | *B. pascuorum* |
| 35 | 71.4 | 14.2 | *B. pascuorum* |
| 36 | 62.5 | 25 | *B. pratorum* |
| 81 | 37.5 | 50 | *B. terrestris* |
| 82 | 50 | 50 | *B. terrestris* |
| 83 | 66.66 | 16.66 | *B. locorum* |
| 84 | 50 | 37.5 | *B. locorum* |
| 86 | 50 | 50 | *B. hypnorum* |

Table S1 presents the individual performance in Experiment 1. Over 85% of the subjects showed a bias towards the allocentric strategy when searching for the reward in the Searching array.

**Experiment 2**

When the model was run including condition, order in which the experimental conditions were presented and location of the reward as fixed factors, the results showed that only condition had an effect on subjects’ performance: condition (estimate *SD* = -1.845, *z* = -5.63, *P* < 0.001, 95% CI = 0.083 to 0.300); order of presentation (estimate *SD* = -0.242, *z* = -0.766, *P* = 0.444, 95% CI = 0.422 to 1.458); location of the reward (estimate *SD* = 0.508, *z* = 1.614, *P* = 0.106, 95% CI = 0.896 to 3.081).

**Table S2.** Individual performance in experiment 2: Learning allocentric and egocentric strategies

| Subject | Allocentric condition | Egocentric condition | Species |
| --- | --- | --- | --- |
| 37 | 83.3 | 50 | *B. bohemicus* |
| 39 | 100 | 16.66 | *B. locorum* |
| 40 | 66.67 | 33.33 | *B. pascuorum* |
| 41 | 100 | 33.33 | *B. pascuorum* |
| 42 | 66.66 | 50 | *B. pascuorum* |
| 45 | 83.33 | 50 | *B. pascuorum* |
| 46 | 66.66 | 33.33 | *B. pascuorum* |
| 49 | 66.66 | 33.33 | *B. pascuorum* |
| 51 | 100 | 0 | *B. pascuorum* |
| 53 | 66.66 | 66.66 | *B. pascuorum* |
| 54 | *83.3* | *50* | *B. pascuorum* |
| 57 | 66.66 | 50 | *B. pascuorum* |
| 61 | 100 | 83.33 | *B. pascuorum* |
| 63 | 83.3 | 20 | *B. pratorum* |
| 65 | *100* | *66.66* | *B. pascuorum* |
| 67 | 83.33 | 33.33 | *B. pratorum* |
| 69 | 100 | 66.66 | *B. pascuorum* |
| 70 | 83.33 | 50 | *B. terrestris* |
| 71 | 100 | 33.33 | *B. hortorum* |
| 77 | *83.33* | *50* | *B. hypnorum* |
| 80 | 83.33 | 33.33 | *B. hypnorum* |

Table S2 presents the individual performance in Experiment 2 for *Allocentric* and *Egocentric* conditions. In italic are the subjects that were not included in the analyses. Whereas all subjects learned to use the allocentric strategy, approximately 43% of them learned to also use the egocentric strategy. These results suggest that the egocentric FoR is also present in bees.

**Figure S2**. Percentage of bees selecting the correct strip during the *Allocentric* (black line) and *Egocentric* (yellow line) condition across the 6 trials in Experiment 1. Dotted grey line indicates chance performance

These results suggest that (1) performance in the *Allocentric* condition was consistently above chance and (2) performance in the *Egocentric* condition tended to slowly improve with experience.

**Experiment 3**

As before, when the model was run including condition, order in which the experimental conditions were presented and location of the reward as fixed factors, the results showed that only condition had an effect on subjects’ performance: condition (estimate *SD* = -1.591, *z* = -3.277, *P* = 0.001, 95% CI = 0.078 to 0.527); order of presentation (estimate *SD* = 0.366, *z* = 0.751, *P* = 0.453, 95% CI = 0.554 to 3.576); location of the reward (estimate *SD* = 0.117, *z* = -0.242, *P* = 0.809, 95% CI = 0.344 to 2.299).

**Table S3.** Individual performance in experiment 2: Learning allocentric and egocentric strategies (II)

| Subject | Allocentric condition | Egocentric condition | Species |
| --- | --- | --- | --- |
| 177 | 100 | 50 | *B. terrestris* |
| 178 | 100 | 50 | *B. terrestris* |
| 197 | 50 | 0 | *B. locorum* |
| 198 | 50 | 50 | *B. locorum* |
| 202 | 50 | 50 | *B. locorum* |
| 203 | 50 | 0 | *B. locorum* |
| 208 | 100 | 0 | *B. locorum* |
| 209 | 50 | 0 | *B. locorum* |
| 216 | 100 | 100 | *B. locorum* |
| 220 | 100 | 0 | *B. pascuorum* |
| 221 | 100 | 0 | *B. pascuorum* |
| 226 | 50 | 50 | *B. terrestris* |
| 227 | 50 | 0 | *B. terrestris* |
| 229 | 100 | 50 | *B. locorum* |
| 233 | 50 | 0 | *B. hortorum* |
| 237 | 50 | 50 | *B. locorum* |
| 242 | 50 | 0 | *B. locorum* |
| 249 | 50 | 50 | *B. terrestris* |
| 251 | 50 | 50 | *B. pascuorum* |
| 257 | 100 | 100 | *B. pascuorum* |

Table S3 presents the individual performance in Experiment 3 for *Allocentric* and *Egocentric* conditions. All subjects correctly chose the strip corresponding to the allocentric strategy in, at least, one experimental trial; 40% did so in both trials. As for the Egocentric condition, 55% of the bees choose the correct strip in, at least, one experimental trial; 10% did so in both trials. These results suggest that the egocentric strategies can also be learned by some bees.

**Figure S3**. Percentage of bees selecting the correct strip during the *Allocentric* (black line) and *Egocentric* (yellow line) conditions across the 6 training trials. Dotted grey line indicates chance performance

As before, these results suggest that performance in the *Allocentric* condition was consistently above chance and performance in the *Egocentric* condition improved across trials.
